# 5G/6G Networks for Wireless Communication and IoT

**DOI:** 10.3390/s26113570

**Published:** 2026-06-04

**Authors:** Chiara Suraci, Giuseppe Araniti

**Affiliations:** Department of Information Engineering, Infrastructure and Sustainable Energy (DIIES), University Mediterranea of Reggio Calabria, 89124 Reggio Calabria, Italy; araniti@unirc.it

## 1. Introduction

Wireless communication systems are experiencing a rapid evolution driven by the increasing demand for intelligent, adaptive, and highly interconnected services. Although fifth-generation (5G) networks have already provided significant improvements in throughput, latency, reliability, and connectivity density compared with previous generations, several emerging applications are pushing research toward the definition of beyond-5G and sixth-generation (6G) paradigms [1]. Future wireless ecosystems are expected to support increasingly heterogeneous and distributed environments, integrating communication, computing, sensing, and artificial intelligence within unified architectures [2].

In this scenario, the Internet of Things (IoT) plays a central role. The growing number of connected devices, together with the emergence of data-intensive services and real-time applications, is progressively transforming wireless networks into intelligent service platforms capable of supporting advanced automation, immersive experiences, and context-aware operations [3]. Emerging technologies such as edge intelligence, distributed artificial intelligence (AI), integrated sensing and communication, Digital Twins, and cloud-to-edge orchestration are expected to become key enablers of future 6G ecosystems [4].

At the same time, several challenges have yet to be satisfactorily addressed. Scalability, energy efficiency, privacy and security are critical issues, particularly in highly dynamic and heterogeneous environments [5]. The integration of AI-based approaches into mobile network ecosystems has also introduced new challenges related to explainability, trustworthiness, and resource optimization [6].

Our Special Issue “5G/6G Networks for Wireless Communication and IoT” aimed to collect recent advances related to next-generation wireless systems and intelligent IoT environments. The published contributions reflect the broad and multidisciplinary nature of current research activities in this field, ranging from communication architectures and intelligent resource management strategies to emerging IoT applications, AI-driven optimization, Digital Twin frameworks, and security solutions.

## 2. Overview of the Contributions

The published articles highlight various lines of research that are currently shaping the evolution of future wireless and IoT systems. The contributions cover a wide range of topics. A brief overview of each one is provided below.

Contribution 1 presents a survey on Symbiotic Radio (SRad) technologies for 6G networks. The article reviews coexistence and resource-sharing mechanisms between active and passive radio systems, discussing approaches based on ambient backscatter communication, cognitive radio, RIS-assisted communications, MIMO techniques, and full-duplex solutions. Possible application scenarios in healthcare, transportation, manufacturing, agriculture, and logistics are also discussed, together with several open issues related to channel estimation, interference management, synchronization, and security.

Contribution 2 addresses resource allocation strategies for Ultra-Reliable Low-Latency Communication (URLLC)/enhanced Mobile Broadband (eMBB) services in 5G IoT environments. The paper proposes a Non-Orthogonal Multiple Access (NOMA)-based model aimed at improving system throughput and spectrum efficiency in the network through the joint optimization of channel selection and power allocation. To address the resulting optimization problem, the authors develop a matching-based channel selection algorithm together with a water-filling power allocation scheme. The simulation results show improvements in throughput and spectral efficiency compared to conventional allocation approaches in heterogeneous service scenarios.

Antenna design solutions for CubeSat applications within the Internet of Space Things (IoST) are presented in Contribution 3. The paper provides a detailed description of a low-profile cognitive radio antenna system that works in the Ultra-High-Frequency (UHF) band. This system is promising because it combines a wideband circularly polarized detection antenna with two antennas that are capable of switching frequencies to enable narrowband communication, all integrated onto the same substrate. This architecture allows CubeSat to perform both detection and communication functions while maintaining the necessary compact size. The experimental results demonstrate the strong performance of the antenna in terms of bandwidth, frequency switching, radio wave behavior, the ability to separate different incoming signals and MIMO operation. All of this contributes to a solution that is well suited for future applications in the IoST, where cognitive radio technology will be available.

Contribution 4 presents a survey which provides an in-depth analysis of the role of 6G in the development of next-generation smart cities. The authors examine scientific works that propose using new technologies such as AI, edge computing, digital twins, terahertz communications, blockchain, and integrated sensing to make cities more interconnected and smart. Furthermore, the article describes various use cases such as smart transportation, law enforcement, nature monitoring, medical care, and 3D services, while also listing technological challenges such as interoperability, energy consumption, and security in highly advanced smart city ecosystems.

Contribution 5 explores resource allocation schemes for NOMA in 5G and future wireless networks, aimed at enhancing spectral efficiency in scenarios of dense IoT and high connectivity. The authors offer a joint channel and power allocation strategy involving a new Channel User Sorting and Filling (CUSF) algorithm combined with water-filling and fractional transmit power control methods. Since NOMA systems present challenges related to user grouping and interference management, this study shows that smart resource optimization can enable the increasing performance demands of 5G/6G communication environments and next-generation IoT services.

The authors of Contribution 6 have discussed the Age-of-Information (AoI) measure in wireless cellular systems, emphasizing its importance for supporting time-critical 5G and IoT services, particularly with regard to massive machine-type communications (mMTC) and URLLC cases. Instead of limiting the discussion to theoretical queueing models, the authors emphasize the discrepancy between current analytical approaches and the actual features of modern wireless networks. They argue that technologies and mechanisms like orthogonal frequency division multiple access (OFDMA), multi-channel random access, correlated devices, and multi-hop communication paths can have a major impact on information freshness in actual deployments. By pointing out the existing limitations and identifying open research areas, their paper offers a valuable perspective on the difficulties 5G/6G and IoT systems will face in ensuring timely and reliable information delivery.

Contribution 7 proposes a processing technique for estimating the direction of arrival (DOA) for next-generation wireless systems, a technique that is becoming increasingly important over time, particularly in key applications such as positioning, sensing, beamforming, and smart IoT services that are expected in 5G/6G networks. This article introduces an approach based on the simultaneous correlative interferometer (CI) capable of jointly utilizing numerous time-domain samples to estimate multiple signal directions, even in crowded and noisy environments. The proposed solution offers particularly high performance in scenarios with a limited number of samples and array elements compared to conventional MUSIC- and CI-based methods. The authors also explore potential applications in dynamic communication scenarios such as large-scale IoT deployments, satellite systems, anti-spoofing mechanisms, and future multi-antenna 6G infrastructures.

Contribution 8 explores new integrated sensing and communication (ISAC) techniques within the context of future evolutionary directions beyond 5G New Radio (NR) systems, which are attracting growing interest due to their suitability for supporting smart and interconnected IoT environments in preparation for sixth-generation wireless networks. In particular, a repeated dual-frequency pulse waveform is proposed for time-division ISAC, in order to enable both sensing and communication services within a single 5G infrastructure that shares the same spectrum resources. Through waveform analysis, vehicle parameter estimation, simulation campaigns, and real-traffic experiments, the authors explore alternatives for uplink configuration. The results highlight how ISAC approaches can integrate reliable sensing capabilities without compromising communication efficiency, thereby justifying their feasibility for next-generation smart mobility and related IoT scenarios.

The authors of Contribution 9 propose advanced wireless communication techniques for systems beyond 5G and 6G, with a particular focus on the integration of smart reconfigurable surfaces capable of simultaneously transmitting and reflecting (STAR-RIS), NOMA, and energy-harvesting methods. The authors propose an innovative STAR-RIS-assisted NOMA framework supported by power beacon energy harvesting, with the goal of improving service interruption probability, ergodic throughput, and average symbol error rate in future wireless networks. The paper presents analytical models for the statistics of the sum of two random variables, critical for the modeling of RIS-assisted channels, and derives closed-form expressions for various performance metrics under both perfect and imperfect Successive Interference Cancellation (SIC) conditions. The presented results demonstrate the advantages of the proposed solution over conventional RIS/NOMA and OMA solutions, showing the potential of STAR-RIS technologies for high-capacity, energy-efficient 6G communication systems.

A further interesting proposal is presented in Contribution 10, which addresses the design of energy-efficient power amplifiers for future 5G/6G communication systems. The paper presents a dynamic load modulation (DLM) power amplifier based on a ferroelectric tunable matching network, aimed at improving efficiency under high peak-to-average power ratio (PAPR) conditions typical of OFDM signals. The experimental results show that the proposed solution achieves more than 50% drain efficiency at 8 dB back-off, while also improving output linearity through adaptive bias modulation techniques.

Contribution 11, which focuses on Orthogonal Time–Frequency Space (OTFS) modulation for beyond-5G and 6G wireless systems, addresses the problem of channel estimation in high-mobility scenarios by proposing advanced solutions. The authors suggest a multi-pilot estimation technique using Constant-Amplitude Zero-Autocorrelation (CAZAC) sequences, which aims to resolve the shortcomings of conventional single-pilot methods about estimation accuracy and dependability. They show that the use of several low-power pilots in delay-Doppler (DD) domain combined with receiver side denoising operations results in enhanced normalized mean square error (NMSE) and bit error rate (BER) performances. In general, through their experiments, the authors illustrate that OTFS modulation is a good candidate for implementation in high-speed communication scenarios.

Contribution 12 is a study on one of the more difficult problems of routing Low-Earth-Orbit (LEO) mega-constellation satellite networks by proposing an intelligent routing setup for beyond-5G and 6G non-terrestrial networks (NTNs). The paper presents a solution based on graph neural networks (GNNs) and deep reinforcement learning (DRL), referred to as GDRL-SFCR, which can optimize end-to-end delay and network load balancing while meeting service function chain (SFC) constraints. By integrating topology-aware learning with AI-driven routing decision making, the proposed approach greatly improves the traffic access success rate and greatly reduces congestion in very dynamic satellite communication scenarios. This highlights the potential of AI-native approaches in a 6G and IoT-enabled space network.

Contribution 13 focuses on random access procedures for 6G satellite–terrestrial integrated communication systems (STICSs), which is a very important feature for enabling massive-scale IoT connectivity in future NTNs. The paper begins by pointing out the shortcomings of conventional fourth-generation (4G)/5G random access preamble designs when they are used in satellite environments, where detection performance can be heavily affected by large propagation delays and carrier frequency offsets (CFOs). As a solution, the authors introduce a CFO-resistant random access preamble design with an exclusive root selection algorithm that not only supports large-scale IoT access but is also compatible with terrestrial network structures. According to simulations, the proposed method is quite robust even under severe satellite communication conditions, making it a good candidate for future integrated 6G wireless ecosystems.

Finally, Contribution 14 explores resource allocation and unmanned aerial vehicle (UAV) trajectory planning in ISAC-enabled vehicular networks. The study suggests a joint optimization method that merges UAV trajectory planning, vehicle association, and subchannel allocation to maximize communication performance while remaining compliant with the sensing and energy requirements. By equipping both UAVs and ground base stations (GBSs) with ISAC features, the suggested structure ensures vehicular communications that are both efficient and characterized by very low latency, which has potential for future 5G/6G and IoT scenarios.

## 3. Conclusions

This Special Issue provides an overview of several research directions that are currently driving the evolution of 5G, beyond-5G, and emerging 6G wireless systems. The published works address heterogeneous yet strongly interconnected topics, including intelligent resource allocation, ISAC technologies, satellite and non-terrestrial networks, AI-driven optimization, IoT architectures, advanced antenna systems, and energy-efficient communication solutions. Overall, the Special Issue highlights how future wireless ecosystems will become increasingly reliant on the integration of communication, sensing, computing, and artificial intelligence to support large-scale and highly dynamic IoT environments. At the same time, the collected contributions confirm that several challenges remain open, particularly in terms of scalability, energy efficiency, interoperability, reliability, and network intelligence. We hope that the published articles will provide useful insights for researchers and practitioners working on next-generation wireless communications and IoT systems while also stimulating further discussion and research activities in these rapidly evolving fields.
